# Exploring the prevalence of reasons adolescents feel unwelcome at school

**DOI:** 10.1371/journal.pone.0346290

**Published:** 2026-05-07

**Authors:** Kristen M. Lucibello, Lin Zheng, Scott T. Leatherdale, Karen A. Patte

**Affiliations:** 1 Department of Health Sciences, Brock University, St. Catharines, Ontario, Canada; 2 School of Public Health Sciences, University of Waterloo, Waterloo, Ontario, Canada; Public Library of Science, UNITED KINGDOM OF GREAT BRITAIN AND NORTHERN IRELAND

## Abstract

Cultivating positive school climates has been recognized as a global and Canadian priority for adolescent health, academic achievement, and development. Feeling welcome at school is an early contributor to overall positive school climate, although detailed understanding of different reasons adolescents may not feel welcome at school at the population-level is limited. The present study examined the prevalence of reasons students felt unwelcome at school and the characteristics of students endorsing each reason. Self-report survey data were used from students (*N* = 15,610, M_age_ ± SD = 15.6 ± 1.2, 48.1% girls) from 41 secondary schools in Ontario, Canada, that participated in the 2022–2023 wave of the COMPASS study. Frequencies calculated the prevalence of each reason for feeling unwelcome at school. Overall, 63.4% of students felt unwelcome at school for at least one reason. Appearance (29.6%), another reason (23.3%), marks at school (19.3%), emotional/psychological challenge (15.7%), and race/ethnicity/culture (10.6%) were the most common reasons for feeling unwelcome at school. Overweight perception, gender diverse identity, and low perceived relative financial affluence were overrepresented in participants that felt unwelcome at school relative to the overall sample. The many reasons adolescents feel unwelcome at school need to be addressed as schools focus on building positive and inclusive school climates, and should be considered across multiple levels (e.g., teacher training, students’ learning materials, policy).

## Introduction

Cultivating positive school climates has been recognized globally and among Canadians as a priority for adolescent health, development, and achievement [1,2]. In line with this priority, researchers have begun to build socioecological frameworks and multi-level approaches to foster more inclusive school environments [3,4]. One important aspect of this positive climate is school belonging, or “the extent to which students feel personally accepted, respected, included, and supported by others in the school social environment” [5]. While school belonging has been associated with higher academic achievement [6], better short and long term mental health [7,8], higher physical activity [9], and less substance use, violence, and poor sexual health [10], declines in school belonging have also been noted among adolescents over time [11]. As such, current surveillance across schools examining upstream contributors to school belonging is essential to inform multi-level prevention and intervention efforts within schools.

Feeling welcome at school is an integral aspect of a positive overall school climate [12,13], and an important early contributor to perceived belonging [14]. Feeling welcome at school can be a global perception, or related to a specific identity factor (e.g., gender, ethnicity, ability) [13,15]. Feeling welcome at school is essential to prioritize for continuing students as well as newcomer students with diverse experiences and identities [16]. Interestingly, while psychosocial (e.g., test anxiety) [17] and identity (e.g., gender, ethnicity) [7,18] correlates of low overall school belonging have been identified, measures have not parsed out factors that are perceived by adolescents to be direct contributors to feeling unwelcome at school. There are numerous identity factors that are influenced at the interpersonal and organizational level that may make adolescents feel unwelcome at school. For example, despite increased awareness and preventative efforts, rates of peer victimization remain stable or even elevated relative to pre-pandemic rates [19], with diverse identity targets including appearance, gender, sexual orientation, race and ethnicity, religion, socioeconomic status, and physical and cognitive ability [20]. Organizational factors may also lead to adolescents feeling unwelcome at school, as increased performance orientation and higher demands within school environments may alienate lower achieving students [21]. School staff can also influence whether the school environment feels welcoming; for example, teachers and sport coaches can perpetuate negative body appearance and weight commentary towards students and in front of their peers [22].

Collectively, this evidence suggests there is an urgent need for understanding the prevalence of specific reasons adolescents feel unwelcome at school among a large population-level sample. This timely data is integral for establishing baseline information to inform and support ongoing school efforts related to school belonging and positive school climates (i.e., school-level policies, teacher/administrator level-training), as well as equity, diversity, and inclusion more broadly. While the school environment can serve as a health and well-being hub [23], inequities can exist within school environments and among students’ experiences of school environments. For example, greater declines in school belonging have been noted among certain population groups over time, including females and adolescents of lower socioeconomic position [24]. As schools can also reflect wider community climates and social hierarchies but provide a context more amenable to change [17], interventions informed by this research may provide protective effects for students that may be more likely to experience discrimination or exclusion outside of the school context. Therefore, the purpose of the present study was to explore the prevalence of different reasons that secondary school students report feeling unwelcome at school. This study will also describe the characteristics of students endorsing different reasons.

## Materials and methods

### Participants and procedures

The current study used cross-sectional student-level survey data from Year 11 (01/09/2022–30/06/2023) of the Cannabis use, Obesity, Mental health, Physical activity, Alcohol use, Smoking, and Sedentary behaviour (COMPASS) study, a large prospective cohort study (2012–2027) of adolescents attending secondary schools across Canada [25]. Schools were purposefully selected based on permitted use of active-information, passive-consent parental permission protocols, which are shown to reduce demands on schools and to increase response rates among youth [26]. Parents/legal guardians received a letter describing the study purpose, perceived risks and benefits, and instruction to contact the research team if they do not consent to their child participating. This protocol protects participating student confidentiality, as student names are not collected unless parents/legal guardians opt to withdraw their child from the study. All adolescents attending participating secondary schools were eligible to participate and could decline participation at any time. Once passive consent was obtained, students who assented to participate self-reported sociodemographic characteristics via the COMPASS student questionnaire during class time. An additional survey item assessed reasons of feeling unwelcome or uncomfortable at school, which was only administered to participating Ontario schools that opted into the Ontario School Climate Module. All procedures received ethical approval from the University of Waterloo Office of Research Ethics (ORE# 30118), Brock University Research Ethics Board (REB #18–099) and appropriate school board committees. Additional information of the COMPASS study can be found in print [25] or online (https://uwaterloo.ca/compass-system).

A total of 18,632 students from Ontario high schools participated in Year 11, of which 3,022 opted not to complete any of the unwelcome/uncomfortable items. Thus, the final sample included 15,610 adolescents across 41 Ontario schools.

### Measures

#### Reasons for feeling unwelcome/uncomfortable at school.

Students were asked to indicate whether they “ever feel unwelcome or uncomfortable at school because of any of the following reasons” (check all that apply, 1 = yes, 0 = no): 1) race, ethnicity, or culture, 2) religion, 3) family isn’t as wealthy/rich as others, 4) gender identity, 5) sexual orientation, 6) image/appearance, 7) an emotional/psychological challenge you may have (e.g., mental health, ADHD), 8) a learning challenge you may have (learning disability), 9) a physical challenge you may have (difficulty hearing, seeing, moving, or speaking), 10) marks at school, and 11) other reasons.

#### Demographic characteristics.

Participants reported their school grade (7–12), perceived family financial comfort relative to their peers at school (more comfortable, as comfortable, and less comfortable), and perceived weight status (very underweight, slightly underweight, about the right weight, slightly overweight, very overweight). Participants also reported their current gender identity (girl/woman, boy/man, non-binary person, two-spirit, I describe my gender differently, and I prefer not to say) and sex assigned at birth (male, female, and I prefer not to say). Three gender categories were derived: cisgender girl/woman (i.e., female and girl/woman), cisgender boy/man (i.e., male and boy/man), and gender diverse (i.e., non-binary, I describe my gender differently, male and girl, female and boy). Finally, participants reported their race and ethnicity, including Asian (East Asian, South Asian, and Southeast Asian), Black, Latino, White, Another ethnicity/multiple ethnicities (Indigenous, Middle Eastern, and Another category), and Do not know/prefer not to answer.

### Data analysis

Descriptive statistics were run for all participant demographic information. The prevalence of each reason for feeling unwelcome/uncomfortable at school was calculated via frequencies. Subsequently, all eleven frequencies were summed to determine the number of reasons students felt unwelcome/uncomfortable at school. Data analyses were conducted in IBM® SPSS Statistics Version 30.

## Results

The final sample was an average of 15.6 years old (SD = 1.2) and was comprised of cisgender girls (48.1%), cisgender boys (46.7%), and gender diverse adolescents (5.2%). The majority of adolescents identified as White (59.9%), followed by Another ethnicity/multiple ethnicities (17.7%), Asian (9.2%), Black (5.2%) and Latino/Latin American (4.0%). Just over half of students perceived themselves as about the right weight (53.6%), and approximately 60% of adolescents considered their family as financially comfortable as their peers. Full sample demographic information can be found in Table 1.

**Table 1:** Demographic characteristics of Ontario secondary school students that participated in the COMPASS study during the 2022/2023 school year (n = 15,610).

	n (%)
Grade	
9	5,018 (32.1)
10	4,247 (27.2)
11	3,504 (22.4)
12	2,841 (18.2)
Gender	
Cis girl/woman	7,334 (48.1)
Cis boy/man	7,110 (46.7)
Gender diverse	793 (5.2)
Race and Ethnicity	
Asian	1,426 (9.2)
Black	802 (5.2)
Latino/Latin American	617 (4.0)
White	9,314 (59.9)
Another ethnicity/multiple ethnicities	2,760 (17.7)
Do not know/prefer not to answer	635 (4.1)
Weight perception	
Underweight	3,051 (19.7)
About the right weight	8,296 (53.6)
Overweight	4,138 (26.7)
Perceived relative financial comfort	
More comfortable	4,569 (29.5)
As comfortable	9,251 (59.7)
Less comfortable	1,676 (10.8)

Over 60% of students reported feeling unwelcome or uncomfortable at school for at least one reason (63.4%), and 30.5% of these students reported two or more reasons. Appearance was the most common reason adolescents reported feeling unwelcome/uncomfortable at school (29.6%), followed by another reason (23.3%), marks at school (19.3%), having an emotional/psychological challenge (15.7%), and race, ethnicity, culture (10.6%). The remaining reasons were reported by under 10% of the sample, and included sexual orientation (7.2%), a learning challenge (6.6%), gender identity (6.0%), family isn’t as wealthy/rich (6.0%), religion (5.5%), and having a physical challenge (5.5%) (Table 2).

**Table 2:** Reasons Ontario secondary school students from Year 11 (2022-2023) of the COMPASS study reported feeling unwelcome and/or uncomfortable at their school (*n* = 15,610).

	n (%)
Felt unwelcome/uncomfortable at school (yes)	
Appearance	4,622 (29.6)
Another reason	3,634 (23.3)
Marks at school	3,010 (19.3)
Emotional/psychological problem	2,444 (15.7)
Race, ethnicity, culture	1,653 (10.6)
Sexual orientation	1,117 (7.2)
A learning challenge	1,037 (6.6)
Gender identity	939 (6.0)
Family isn’t as wealthy/rich	939 (6.0)
Religion	854 (5.5)
A physical challenge	851 (5.5)
Number of reasons	
0 reasons	5,712 (36.6)
1 reason	5,144 (33.0)
2 reasons	1,978 (12.7)
3 or more reasons	2,776 (17.8)

Consistent with our overall sample characteristics, students who reported the top five reasons were mostly cisgenders girls, followed by cisgender boys. While gender diverse adolescents were least represented across the five reasons, they were overrepresented (e.g., 8.8% of appearance reason, 16.6% of emotional/psychological problem) relative to the overall sample (5.2% gender diverse). Similarly, overweight perceptions were largely overrepresented across the five categories (e.g., 39.9% of appearance reason, 34.5% marks at school) relative to the overall sample (26.7%). Like the overall sample, perceiving oneself as comparably financially comfortable as peers was most common across categories, although perceived less financial comfort was overrepresented (e.g., 21.5% for psychological/emotional problems) relative to the overall sample (10.8%). Finally, Asian, Black, and adolescents with multiple ethnicities were overrepresented in feeling unwelcome/uncomfortable at school due to race/ethnicity/culture. Grade distributions were consistent between the overall sample and the top five reasons (percentage of group make up decreases as grade increases). Full descriptive statistics across each reason for feeling unwelcome/uncomfortable at school can be found in Tables 3 and 4.

**Table 3:** Demographic features of adolescents from the COMPASS study who report feeling unwelcome/uncomfortable at school for the five most commonly reported reasons (reported by over 10% of students).

	Appearance *n =* 4,622	Another reason *n =* 3,634	Marks at school *n =* 3,010	Emotional/ psychological problem *n =* 2,444	Race/ ethnicity/culture *n =* 1,653
Gender					
Cis girl	2,618 (56.6%)	1,828 (50.3%)	1,673 (55.6%)	1,342 (54.9%)	789 (47.7%)
Cis boy	1,473 (31.9%)	1,487 (40.9%)	1,013 (33.7%)	591 (24.2%)	664 (40.2%)
Gender diverse	409 (8.8%)	206 (5.7%)	238 (7.9%)	406 (16.6%)	147 (8.9%)
Missing	122 (2.6%)	113 (3.1%)	86 (2.9%)	105 (4.3%)	53 (3.2%)
Grade					
9	1,562 (33.8%)	1,286 (35.4%)	916 (30.4%)	680 (27.8%)	490 (29.6%)
10	1,255 (27.2%)	1,019 (28.0%)	788 (26.2%)	658 (26.9%)	421 (25.5%)
11	1,021 (22.1%)	784 (21.6%)	710 (23.6%)	604 (24.7%)	408 (24.7%)
12	784 (17.0%)	545 (15.0%)	596 (19.8%)	502 (20.5%)	334 (20.2%)
Missing	–	–	–	–	–
Race and ethnicity					
Do not know/prefer not to answer	209 (4.5%)	231 (6.4%)	138 (4.6%)	144 (5.9%)	76 (4.6%)
Asian	455 (9.8%)	274 (7.5%)	292 (9.7%)	137 (5.6%)	406 (24.6%)
Latino/Latin American	163 (3.5%)	158 (4.3%)	125 (4.2%)	77 (3.2%)	118 (7.1%)
Black	214 (4.6%)	192 (5.3%)	144 (4.8%)	83 (3.4%)	305 (18.5%)
Another ethnicity/multiple ethnicities	980 (21.2%)	651 (17.9%)	591 (19.6%)	545 (22.3%)	543 (32.8%)
White	2,594 (56.1%)	2,117 (58.3%)	1,714 (56.9%)	1,454 (59.5%)	201 (12.2%)
Missing	7 (0.2%)	11 (0.3%)	6 (0.2%)	4 (0.2%)	4 (0.2%)
Weight perception					
Underweight	920 (19.9%)	745 (20.5%)	599 (19.9%)	538 (22.0%)	357 (21.6%)
Overweight	1,845 (39.9%)	974 (26.8%)	1,038 (34.5%)	893 (36.5%)	502 (30.4%)
About right	1,827 (39.5%)	1,887 (51.9%)	1,356 (45.0%)	994 (40.7%)	777 (47.0%)
Missing	30 (0.6%)	28 (0.8%)	17 (0.6%)	19 (0.8%)	17 (1.0%)
Financial comfort					
More comfortable	1,096 (23.7%)	995 (27.4%)	706 (23.5%)	539 (22.1%)	458 (27.7%)
As comfortable	2,679 (58.0%)	2,174 (59.8%)	1,748 (58.1%)	1,361 (55.7%)	935 (56.6%)
Less comfortable	832 (18.0%)	436 (12.0%)	538 (17.9%)	526 (21.5%)	252 (15.2%)
Missing	15 (0.3%)	29 (0.8%)	18 (0.6%)	18 (0.7%)	8 (0.5%)

*Note.* Survey data from students that participated in the 2022/2023 school year at participating COMPASS secondary schools in Ontario.

**Table 4:** Demographic features of adolescents from the COMPASS study who report feeling unwelcome/uncomfortable at school for different reasons (reasons reported by under 10% of students).

	Sexual orientation *n =* 1,117	A learning challenge *n =* 1,037	Gender identity *n =* 939	Family isn’t as wealthy/rich *n =* 939	Religion *n* = 854	A physical challenge *n =* 851
Gender						
Cis girl	444 (39.7%)	450 (43.4%)	312 (33.2%)	504 (53.7%)	384 (45.0%)	330 (38.8%)
Cis boy	219 (19.6%)	330 (31.8%)	131 (14.0%)	299 (31.8%)	332 (38.9%)	299 (35.1%)
Gender diverse	376 (33.7%)	207 (20.0%)	422 (44.9%)	106 (11.3%)	112 (13.1%)	179 (21.0%)
Missing	78 (7.0%)	50 (4.8%)	74 (7.9%)	30 (3.2%)	26 (3.0%)	43 (5.1%)
Grade						
9	278 (24.9%)	310 (29.9%)	243 (25.9%)	283 (30.1%)	239 (28.0%)	281 (33.0%)
10	311 (27.8%)	281 (27.1%)	245 (26.1%)	242 (25.8%)	227 (26.6%)	226 (26.6%)
11	270 (24.2%)	255 (24.6%)	245 (26.1%)	206 (21.9%)	215 (25.2%)	193 (22.7%)
12	258 (23.1%)	191 (18.4%)	206 (21.9%)	208 (22.2%)	173 (20.3%)	151 (17.7%)
Missing	–	–	–	–	–	–
Race and ethnicity						
Do not know/prefer not to answer	73 (6.5%)	84 (8.1%)	72 (7.7%)	46 (4.9%)	49 (5.7%)	73 (8.6%)
Asian	76 (6.8%)	64 (6.2%)	71 (7.6%)	115 (12.2%)	114 (13.3%)	89 (10.5%)
Latino/Latin American	36 (3.2%)	29 (2.8%)	29 (3.1%)	58 (6.2%)	30 (3.5%)	35 (4.1%)
Black	51 (4.6%)	61 (5.9%)	56 (6.0%)	86 (9.2%)	75 (8.8%)	48 (5.6%)
Another ethnicity/multiple ethnicities	229 (20.5%)	233 (22.5%)	200 (21.3%)	232 (24.7%)	261 (30.6%)	221 (26.0%)
White	652 (58.4%)	564 (54.4%)	510 (54.3%)	401 (42.7%)	321 (37.6%)	385 (45.2%)
Missing	–	2 (0.2%)	1 (0.1%)	1 (0.1%)	4 (0.5%)	–
Weight perception						
Underweight	231 (20.7%)	259 (25.0%)	201 (21.4%)	223 (23.7%)	194 (22.7%)	212 (24.9%)
Overweight	412 (36.9%)	365 (35.2%)	351 (37.4%)	348 (37.1%)	272 (31.9%)	324 (38.1%)
About right	465 (41.6%)	408 (39.3%)	378 (40.3%)	365 (38.9%)	380 (44.5%)	306 (36.0%)
Missing	9 (0.8%)	5 (0.5%)	9 (1.0%)	3 (0.3%)	8 (0.9%)	9 (1.1%)
Financial comfort						
More comfortable	269 (24.1%)	220 (21.2%)	217 (23.1%)	102 (10.9%)	265 (31.0%)	185 (21.7%)
As comfortable	626 (56.0%)	591 (57.0%)	515 (54.8%)	408 (43.5%)	447 (52.3%)	479 (56.3%)
Less comfortable	218 (19.5%)	218 (21.0%)	200 (21.3%)	424 (45.2%)	137 (16.0%)	182 (21.4%)
Missing	4 (0.4%)	8 (0.8%)	7 (0.7%)	5 (0.5%)	5 (0.6%)	5 (0.6%)

*Note.* Survey data from students that participated in the 2022/2023 school year at participating COMPASS secondary schools in Ontario.

## Discussion

The present study examined the reasons high school students reported for feeling unwelcome at school and their associated demographic characteristics. Over 60% of students felt uncomfortable at school for at least one reason, with approximately half of these students reporting more than one reason. The diverse reasons students reported for feeling unwelcome or uncomfortable at school were largely in line with previous evidence [20]. Despite increased attention towards school climate, bullying prevention, and equity, diversity, and inclusion within schools, a high prevalence of students feel unwelcome at school. These results have important intervention and policy implications for school belonging, and the downstream health, developmental, and academic outcomes of youth.

Appearance was the most common reason adolescents reported feeling unwelcome or uncomfortable at school. This is consistent with ample evidence highlighting the salience of appearance among adolescents [27] and declines in body image noted among this age group [28]. In addition, the increasingly stringent and complex body ideals perpetuated on social media may exacerbate appearance culture and concerns, as well as body surveillance among peers [29]. Peer relationships are considered important influences on perceived school belonging by both students and teachers [30], and the appearance culture of adolescence may be difficult to monitor and disrupt in the school context. However, teachers and schools are perceived to play an important role in cultivating physical and psychological safety at school by thwarting bullying or negative interpersonal interactions [30]. Contextualized to appearance reasons for feeling unwelcome at school, it is important that school contexts de-emphasize appearance (e.g., policy prohibiting weight commentary between students, teachers should not make disparaging remarks about their own appearance) and focus on skill development, self-efficacy and fostering functional views of the self [31]. While numerous aspects of appearance could be driving feeling unwelcome at school, it is important to note that adolescents with overweight perceptions were overrepresented in feeling unwelcome/uncomfortable at school due to appearance, which aligns with the weight bias consistently noted within school contexts (i.e., physical education) [32] and among educators [22,33]. As such, training and policy creation in this area needs to include weight inclusive experts and promote appearance diversity and inclusivity as opposed to appearance management and manipulation to conform to body and appearance ideals.

Interestingly, ‘another reason’ was the second most identified reason adolescents felt unwelcome or uncomfortable at school. Similarly, adolescents have qualitatively reported numerous infrequent or unique targets of peer victimization that were not considered part of broader coded categories, including niche personal interests, group affiliations (e.g., a girl on the boys’ football team) or stepping in when a classmate was being bullied [20]. These specific reasons further reiterate the complexity of creating positive school climates and the individual needs of students. At a broader level, this finding has strong implications for implementing zero tolerance policies and regular surveillance around reasons for victimization or feeling uncomfortable at school. More specifically, it is essential to understand these additional reasons as research continues to build and test policy and practice around more positive school climates, to ensure undetected or overlooked reasons are adequately identified and in turn addressed. Qualitative and participatory approaches would help to better understand the needs of students as well as capture their nuanced and unique experiences [34,35].

Marks at school was another common reason students reported feeling unwelcome or uncomfortable at school. Students have identified that the high pressures and extensive homework and testing they experience through school is stressful [30] and perceived school pressure has increased over time [36]; an increased demand for high performance within schools could potentially promote feeling unwelcome among lower performing students [21]. Importantly, a positive feedback loop of feeling unwelcome at school due to low marks could in turn relate to more absenteeism and reduced academic performance [37] which in turn would further exacerbate feeling unwelcome at school due to marks. As academic pressure continues to be acknowledged globally as a contributor to poor adolescent health [38,39], interventions to improve feeling welcome at school regardless of marks may be particularly relevant for perceived support from teachers and positive teacher-student relationships [40].

Finally, feeling unwelcome at school due to psychological/emotional problems was reported by just over 15% of the sample. Unlike more visually-motivated reasons for feeling unwelcome at school (e.g., appearance), it is unclear whether adolescents who feel unwelcome at school due to psychological/emotional problems have disclosed these problems to peers and/or teachers or are concealing them due to perceived social stigma. While recent attempts to increase mental health literacy and prevent stigma through school-based interventions have been noted [41], biases (personal or perceived) can still be held among adolescents [42]. While school resources can be an important first avenue of support for adolescents experiencing psychological/emotional problems, students with depression and anxiety are particularly reluctant to seek help for their mental health at school [43]. Thus, those with psychological/emotional problems whose schools and/or experiences at school lead them to feel unwelcome because of these problems may be even less likely to access resources or seek support [44]. Long-term, multi-level education and policy recommendations including integrating mental health education into curriculum, training for educators and staff to identify mental health concerns, and easily available resources and help-seeking infrastructure [45]) may better support and help to alleviate feelings of being unwelcome at school due to psychological/emotional problems.

While only assessed descriptively, some interesting patterns were noted with respect to the identity factors of students reporting feeling unwelcome or uncomfortable at school for different reasons. Certain identity features were consistent with previous research and the reason adolescents felt uncomfortable at school, including adolescents with overweight perceptions being overrepresented across appearance reasons, and Asian, Black, and adolescents with multiple ethnic identities being overrepresented within race, ethnicity, and cultural reasons for feeling unwelcome [22,46]. However, other reasons for feeling unwelcome at school were not directly represented by the identity factor, although they may still be related. For example, gender diverse adolescents and adolescents with low financial comfort relative to peers were overrepresented among those reporting emotional/psychological problems as reasons for feeling unwelcome at school. Higher rates of emotional/psychological problems have been consistently noted among gender diverse adolescents [47] and adolescents with low socioeconomic status [48]. Similarly, adolescents with overweight perceptions reporting feeling unwelcome at school due to grades, and bias against higher-weight adolescents in terms of their perceived academic ability and grades is common among teachers [33]. Finally, gender diverse adolescents were also overrepresented within feeling unwelcome at school due to appearance, which may highlight their creative and potential non gender conforming expression through appearance [49]. These findings underscore the importance of extending intervention efforts beyond identities that are directly targeted through interpersonal interactions or policy messaging, by also understanding the downstream effects of certain identities which may be more vulnerable to multiple, concurrent reasons for feeling unwelcome or uncomfortable at school. Ensuring comprehensive support is particularly crucial for fostering the health, well-being, and academic success of groups that may already be at greater risk for negative outcomes or poorer experiences within school environments.

Additional reasons for feeling unwelcome or uncomfortable at school were each noted by under 10% of the sample, including sexual orientation, a learning challenge, gender identity, perceived family wealth, religion, and a physical challenge. Despite the smaller prevalence compared to other reported reasons, it is also important to consider the proportion of adolescents who would endorse these identity factors, and thus be vulnerable to feeling unwelcome or uncomfortable at school because of it. For example, 10.8% of students perceived their family to be less financially comfortable than their peers; 6.0% of the sample in turn felt unwelcome or uncomfortable at school because their family isn’t as wealthy. Similarly, 5.2% of the sample reported a gender diverse identity, whereas 6% reported that their gender identity (which could include cisgender or gender diverse adolescents) was a reason they felt unwelcome or uncomfortable at school. This suggests that a high proportion of adolescents who are a part of these smaller subgroups are endorsing feeling unwelcome or uncomfortable at school for that reason. This may in part be due to a lack of perceived similar others, as well as a lack of perceived acceptance demonstrated through school infrastructure (e.g., sexual majority/minority alliances) [50]. As such, these reasons for feeling unwelcome or uncomfortable at school still reflect important barriers that should be acknowledged and considered within school decision making to improve school belonging. Further research should also examine how feeling unwelcome at school for different reasons may relate to and vary as a function of individual-level (i.e., school connectedness, anxiety, bullying) and school-level (i.e., size, median household income) factors.

Study findings reinforce the importance of schools prioritizing how to cultivate positive school climates, while highlighting the complicated and intersectional influences that may contribute to this lack of perceived belonging. Considering that feeling or anticipating threat due to one’s social identity can negatively impact academic engagement [51,52] and even physical and mental health [53], feeling welcome at school is foundational for positive downstream educational and health outcomes of adolescents. The reasons adolescents feel unwelcome at school can inform person-level influences (i.e., teacher and peer support) and policy and system-level influences (i.e., staff professional development, school policy) that function within the broader political and cultural landscape the school exists within (i.e., social climate, government education reform) [3]. While more obvious and explicit forms of exclusion or bias may be easier to identify (i.e., ensuring different identity factors are protected under school anti-bullying policies), ensuring diverse voices and experiences are enlisted in the socioecological reform process will support schools in identifying more implicit and engrained forms of exclusion or bias within the school system (i.e., inclusive language in school policies, diverse and representative imagery within school materials). It is also essential that adolescents be involved in decision making surrounding positive school climates, as adolescents recommend that schools establish better communication and inclusion of their voices in policy decisions and protocols that impact them [54]. This is consistent with Article 12 in the United Nations Convention on the Rights of the Child (UNCRC), such that adolescents have a right to the opportunity to voice their opinions and have them taken seriously with matters that impact them. It is also consistent with participatory research which reiterates that adolescents are experts in their own lived experiences [35]. By meaningfully engaging adolescents with diverse experiences throughout the decision-making process, schools may generate highly relevant and impactful policies and training to enhance feeling welcome at school and in turn school belonging among adolescents [34,55].

### Strengths and limitations

This study has notable strengths and limitations to address. With respect to strengths, understanding the diversity and prevalence of reasons adolescents feel unwelcome at school furthers the national priority of fostering more positive school climates, while also elucidating high risk subgroups for feeling unwelcome at school who may disproportionately benefit from various levels of intervention. In addition to the large sample size, the distributions of sample characteristics among the COMPASS participants are consistent with nationally representative databases [56]. Finally, the use of an active-information, passive-consent permission protocol reduces social desirability bias, protects confidentiality, and reduces underreporting [26].

With respect to limitations, the reason of ‘another reason’ for feeling unwelcome or uncomfortable at school was closed ended, and thus participants were unable to report their specific reason. Since it was the second most selected reason, further research is imperative to better understand what reasons adolescents were referring to. Furthermore, this survey was only administered in Ontario. As such, whether and how these reasons for feeling unwelcome at school generalize to different Canadian regions cannot be determined. Additionally, the available data makes it difficult to determine what experiences or sources led to adolescents feeling unwelcome or uncomfortable at school for any given reason (e.g., cyberbullying from peers, messaging from teachers and/or administrators), which limits targeted and empirically informed interventions to foster more positive school climates. Finally, given the study’s cross-sectional nature causality cannot be established.

## Conclusion

Over 60% of adolescents reported feeling unwelcome or uncomfortable at school for at least one reason. The most common reasons were appearance, another reason, marks at school, psychological/emotional problems, and race, culture, and ethnicity. Given that positive school climates are a global priority and a robust predictor of numerous health and well-being outcomes, this research may help identify vulnerable subgroups as well as inform socioecological frameworks and interventions that combine policy change and school-level training to try and foster more welcoming and inclusive school climates for high school students.
